# 1-(1,3-Benzodioxol-5-yl)butan-1-one

**DOI:** 10.1107/S1600536808040300

**Published:** 2008-12-20

**Authors:** Wei Cheng, Ran Lv, Hong-Jun Zhu

**Affiliations:** a Department of Applied Chemistry, College of Science, Nanjing University of Technology, Nanjing 210009, People’s Republic of China

## Abstract

In the mol­ecule of the title compound, C_11_H_12_O_3_, the dioxole ring adopts an envelope conformation. In the crystal structure, weak inter­molecular C—H⋯O hydrogen bonds link the mol­ecules into chains.

## Related literature

For general background, see: Nichols (1986 ▶). For a related structure, see: Zhu (2003 ▶). For bond-length data, see: Allen *et al.* (1987 ▶).
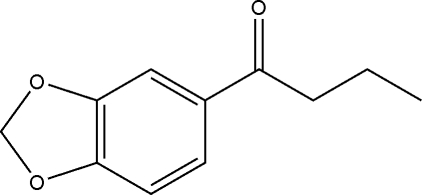


## Experimental

### 

#### Crystal data

C_11_H_12_O_3_
*M* *_r_* = 192.21
Monoclinic, 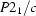

*a* = 11.944 (2) Å
*b* = 11.143 (2) Å
*c* = 7.4600 (15) Å
β = 100.69 (3)°
*V* = 975.6 (3) Å^3^
*Z* = 4
Mo *K*α radiation
μ = 0.10 mm^−1^
*T* = 298 (2) K
0.30 × 0.20 × 0.10 mm

#### Data collection

Enraf–Nonius CAD-4 diffractometer
Absorption correction: ψ scan (North *et al.*, 1968 ▶) *T*_min_ = 0.972, *T*_max_ = 0.991
1869 measured reflections
1775 independent reflections
1166 reflections with *I* > 2σ(*I*)
*R*_int_ = 0.045
3 standard reflections frequency: 120 min intensity decay: 1%

#### Refinement

*R*[*F* ^2^ > 2σ(*F* ^2^)] = 0.070
*wR*(*F* ^2^) = 0.174
*S* = 1.01
1775 reflections
127 parameters
H-atom parameters constrained
Δρ_max_ = 0.27 e Å^−3^
Δρ_min_ = −0.30 e Å^−3^

### 

Data collection: *CAD-4 Software* (Enraf–Nonius, 1985 ▶); cell refinement: *CAD-4 Software*; data reduction: *XCAD4* (Harms & Wocadlo, 1995 ▶); program(s) used to solve structure: *SHELXS97* (Sheldrick, 2008 ▶); program(s) used to refine structure: *SHELXL97* (Sheldrick, 2008 ▶); molecular graphics: *PLATON* (Spek, 2003 ▶); software used to prepare material for publication: *SHELXTL* (Sheldrick, 2008 ▶) and *PLATON*.

## Supplementary Material

Crystal structure: contains datablocks I, global. DOI: 10.1107/S1600536808040300/hk2590sup1.cif

Structure factors: contains datablocks I. DOI: 10.1107/S1600536808040300/hk2590Isup2.hkl

Additional supplementary materials:  crystallographic information; 3D view; checkCIF report

## Figures and Tables

**Table 1:** Hydrogen-bond geometry (Å, °)

*D*—H⋯*A*	*D*—H	H⋯*A*	*D*⋯*A*	*D*—H⋯*A*
C9—H9*A*⋯O1^i^	0.93	2.53	3.209 (4)	130

Symmetry code: (i) 
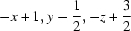
.

**Fig. 1.**
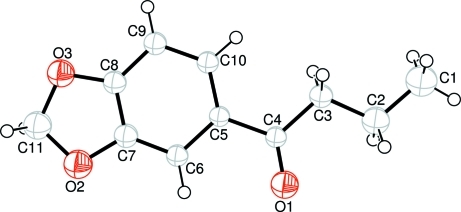
The molecular structure of the title molecule, with the atom-numbering scheme.

**Fig. 2.**
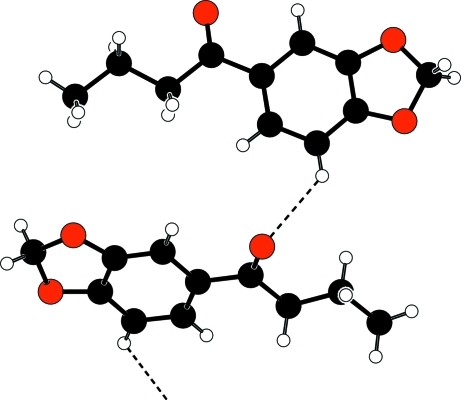
A partial packing diagram of the title compound. Hydrogen bonds are shown as dashed lines.

## supplementary crystallographic information

### Comment

The title compound is an important medicine intermediate used to synthesize 3,4-methylenedioxy-alpha-ethyl-*N*-methylphenethylamine, which is a lesser-known hallucinogenic phenethylamine (Nichols, 1986). We report herein its crystal structure, which is of interest to us in the field.

In the molecule of title compound (Fig. 1), the bond lengths (Allen *et al.*,  1987) and angles are within normal ranges. Ring B (C5–C10) is, of course, planar, while ring A (O2/O3/C7/C8/C11) adopts envelope conformation with C11 atom displaced by 0.147 (3) Å from the plane of the other ring atoms. Atoms O1, C3 and C4 are -0.032 (3), 0.050 (3) and 0.044 (3) Å away from the plane of the benzene ring.

In the crystal structure, weak intermolecular C—H···O hydrogen bonds (Table 1) link the molecules into chains (Fig. 2), in which they may be effective in the stabilization of the structure.

### Experimental

The title compound was synthesized according to a literature method (Zhu, 2003). Crystals suitable for X-ray analysis were obtained by dissolving the title compound (0.3 g) in ethanol (25 ml), and evaporating the solvent slowly at room temperature for about 4 d.

### Refinement

H atoms were positioned geometrically, with C—H = 0.93, 0.97 and 0.96 Å for aromatic, methylene and methyl H, respectively, and constrained to ride on their parent atoms with U_iso_(H) = xU_eq_(C), where x = 1.5 for methyl H and x = 1.2 for all other H atoms.

### Crystal data

**Table:**

C_11_H_12_O_3_	*F*(000) = 408
*M**_r_* = 192.21	*D*_x_ = 1.309 Mg m^−^^3^
Monoclinic, *P*2_1_/*c*	Mo *K*α radiation, λ = 0.71073 Å
Hall symbol: -P 2ybc	Cell parameters from 25 reflections
*a* = 11.944 (2) Å	θ = 9–12°
*b* = 11.143 (2) Å	µ = 0.10 mm^−^^1^
*c* = 7.4600 (15) Å	*T* = 298 K
β = 100.69 (3)°	Block, colourless
*V* = 975.6 (3) Å^3^	0.30 × 0.20 × 0.10 mm
*Z* = 4	

### Data collection

**Table:**

Enraf–Nonius CAD-4 diffractometer	1166 reflections with *I* > 2σ(*I*)
Radiation source: fine-focus sealed tube	*R*_int_ = 0.045
graphite	θ_max_ = 25.3°, θ_min_ = 1.7°
ω/2θ scans	*h* = 0→14
Absorption correction: ψ scan (North *et al.*, 1968)	*k* = 0→13
*T*_min_ = 0.972, *T*_max_ = 0.991	*l* = −8→8
1869 measured reflections	3 standard reflections every 120 min
1775 independent reflections	intensity decay: 1%

### Refinement

**Table:**

Refinement on *F*^2^	Primary atom site location: structure-invariant direct methods
Least-squares matrix: full	Secondary atom site location: difference Fourier map
*R*[*F*^2^ > 2σ(*F*^2^)] = 0.070	Hydrogen site location: inferred from neighbouring sites
*wR*(*F*^2^) = 0.174	H-atom parameters constrained
*S* = 1.00	*w* = 1/[σ^2^(*F*_o_^2^) + (0.05*P*)^2^ + 2.1*P*] where *P* = (*F*_o_^2^ + 2*F*_c_^2^)/3
1775 reflections	(Δ/σ)_max_ < 0.001
127 parameters	Δρ_max_ = 0.27 e Å^−^^3^
0 restraints	Δρ_min_ = −0.30 e Å^−^^3^

### Special details

**Table:**

Geometry. All e.s.d.'s (except the e.s.d. in the dihedral angle between two l.s. planes) are estimated using the full covariance matrix. The cell e.s.d.'s are taken into account individually in the estimation of e.s.d.'s in distances, angles and torsion angles; correlations between e.s.d.'s in cell parameters are only used when they are defined by crystal symmetry. An approximate (isotropic) treatment of cell e.s.d.'s is used for estimating e.s.d.'s involving l.s. planes.
Refinement. Refinement of *F*^2^ against ALL reflections. The weighted *R*-factor *wR* and goodness of fit *S* are based on *F*^2^, conventional *R*-factors *R* are based on *F*, with *F* set to zero for negative *F*^2^. The threshold expression of *F*^2^ > σ(*F*^2^) is used only for calculating *R*-factors(gt) *etc*. and is not relevant to the choice of reflections for refinement. *R*-factors based on *F*^2^ are statistically about twice as large as those based on *F*, and *R*- factors based on ALL data will be even larger.

### Fractional atomic coordinates and isotropic or equivalent isotropic displacement parameters (Å^2^)

**Table:**

	*x*	*y*	*z*	*U*_iso_*/*U*_eq_	
O1	0.4098 (2)	0.2566 (2)	0.9257 (3)	0.0479 (7)	
O2	0.8478 (2)	0.2368 (2)	1.0244 (4)	0.0521 (7)	
O3	0.8912 (2)	0.0624 (2)	0.8880 (3)	0.0475 (7)	
C1	0.1275 (3)	0.0544 (4)	0.6397 (6)	0.0625 (12)	
H1A	0.0552	0.0930	0.6344	0.094*	
H1B	0.1286	−0.0198	0.7055	0.094*	
H1C	0.1394	0.0384	0.5181	0.094*	
C2	0.2211 (3)	0.1353 (3)	0.7358 (6)	0.0486 (10)	
H2A	0.2172	0.2110	0.6708	0.058*	
H2B	0.2075	0.1518	0.8576	0.058*	
C3	0.3397 (3)	0.0849 (3)	0.7510 (4)	0.0347 (8)	
H3A	0.3543	0.0698	0.6293	0.042*	
H3B	0.3437	0.0086	0.8145	0.042*	
C4	0.4319 (3)	0.1669 (3)	0.8506 (4)	0.0284 (7)	
C5	0.5527 (2)	0.1323 (3)	0.8527 (4)	0.0268 (7)	
C6	0.6389 (3)	0.2119 (3)	0.9436 (4)	0.0303 (7)	
H6A	0.6211	0.2819	1.0002	0.036*	
C7	0.7489 (3)	0.1792 (3)	0.9424 (4)	0.0362 (8)	
C8	0.7769 (3)	0.0757 (3)	0.8658 (4)	0.0342 (8)	
C9	0.6943 (3)	−0.0028 (3)	0.7756 (4)	0.0332 (8)	
H9A	0.7136	−0.0722	0.7190	0.040*	
C10	0.5818 (3)	0.0280 (3)	0.7748 (4)	0.0301 (7)	
H10A	0.5243	−0.0235	0.7199	0.036*	
C11	0.9378 (3)	0.1710 (3)	0.9731 (6)	0.0503 (10)	
H11A	0.9952	0.1528	1.0796	0.060*	
H11B	0.9731	0.2175	0.8887	0.060*	

### Atomic displacement parameters (Å^2^)

**Table:**

	*U*^11^	*U*^22^	*U*^33^	*U*^12^	*U*^13^	*U*^23^
O1	0.0483 (15)	0.0414 (15)	0.0506 (16)	0.0091 (11)	0.0003 (12)	−0.0123 (13)
O2	0.0351 (13)	0.0524 (16)	0.0651 (18)	−0.0039 (12)	−0.0004 (12)	−0.0165 (14)
O3	0.0378 (14)	0.0530 (16)	0.0520 (16)	0.0046 (11)	0.0089 (11)	−0.0057 (13)
C1	0.039 (2)	0.078 (3)	0.066 (3)	−0.006 (2)	−0.0016 (19)	−0.017 (2)
C2	0.048 (2)	0.041 (2)	0.053 (2)	−0.0024 (17)	−0.0016 (17)	−0.0038 (18)
C3	0.0429 (19)	0.0340 (18)	0.0241 (17)	−0.0047 (14)	−0.0021 (14)	−0.0017 (14)
C4	0.0428 (18)	0.0209 (15)	0.0211 (15)	0.0085 (13)	0.0051 (13)	−0.0006 (13)
C5	0.0310 (16)	0.0301 (17)	0.0180 (15)	−0.0011 (13)	0.0015 (12)	0.0033 (13)
C6	0.0362 (17)	0.0267 (16)	0.0292 (17)	0.0033 (13)	0.0095 (13)	−0.0073 (13)
C7	0.0361 (18)	0.0399 (19)	0.0306 (18)	−0.0056 (15)	0.0016 (14)	−0.0005 (15)
C8	0.0432 (19)	0.0326 (18)	0.0262 (17)	0.0066 (14)	0.0043 (14)	−0.0014 (14)
C9	0.0402 (19)	0.0294 (17)	0.0309 (17)	0.0037 (14)	0.0093 (14)	−0.0076 (14)
C10	0.0425 (19)	0.0259 (16)	0.0207 (15)	−0.0017 (13)	0.0030 (13)	−0.0028 (13)
C11	0.041 (2)	0.053 (2)	0.057 (2)	−0.0099 (18)	0.0085 (17)	−0.004 (2)

### Geometric parameters (Å, °)

**Table:**

O1—C4	1.200 (4)	C3—H3B	0.9700
O2—C7	1.384 (4)	C4—C5	1.491 (4)
O2—C11	1.410 (4)	C5—C10	1.372 (4)
O3—C8	1.353 (4)	C5—C6	1.432 (4)
O3—C11	1.431 (4)	C6—C7	1.364 (4)
C1—C2	1.510 (5)	C6—H6A	0.9300
C1—H1A	0.9600	C7—C8	1.356 (5)
C1—H1B	0.9600	C8—C9	1.395 (4)
C1—H1C	0.9600	C9—C10	1.385 (4)
C2—C3	1.508 (5)	C9—H9A	0.9300
C2—H2A	0.9700	C10—H10A	0.9300
C2—H2B	0.9700	C11—H11A	0.9700
C3—C4	1.515 (4)	C11—H11B	0.9700
C3—H3A	0.9700		
			
C7—O2—C11	105.7 (3)	C10—C5—C4	122.4 (3)
C8—O3—C11	105.2 (3)	C6—C5—C4	117.0 (3)
C2—C1—H1A	109.5	C7—C6—C5	116.1 (3)
C2—C1—H1B	109.5	C7—C6—H6A	122.0
H1A—C1—H1B	109.5	C5—C6—H6A	122.0
C2—C1—H1C	109.5	C8—C7—C6	122.9 (3)
H1A—C1—H1C	109.5	C8—C7—O2	108.9 (3)
H1B—C1—H1C	109.5	C6—C7—O2	128.1 (3)
C3—C2—C1	114.6 (3)	O3—C8—C7	111.3 (3)
C3—C2—H2A	108.6	O3—C8—C9	126.7 (3)
C1—C2—H2A	108.6	C7—C8—C9	121.9 (3)
C3—C2—H2B	108.6	C10—C9—C8	116.4 (3)
C1—C2—H2B	108.6	C10—C9—H9A	121.8
H2A—C2—H2B	107.6	C8—C9—H9A	121.8
C2—C3—C4	113.5 (3)	C5—C10—C9	122.0 (3)
C2—C3—H3A	108.9	C5—C10—H10A	119.0
C4—C3—H3A	108.9	C9—C10—H10A	119.0
C2—C3—H3B	108.9	O2—C11—O3	107.9 (3)
C4—C3—H3B	108.9	O2—C11—H11A	110.1
H3A—C3—H3B	107.7	O3—C11—H11A	110.1
O1—C4—C5	120.5 (3)	O2—C11—H11B	110.1
O1—C4—C3	121.9 (3)	O3—C11—H11B	110.1
C5—C4—C3	117.6 (3)	H11A—C11—H11B	108.4
C10—C5—C6	120.6 (3)		
			
C1—C2—C3—C4	−179.1 (3)	C11—O3—C8—C7	−4.7 (4)
C2—C3—C4—O1	7.5 (5)	C11—O3—C8—C9	174.6 (3)
C2—C3—C4—C5	−172.6 (3)	C6—C7—C8—O3	−178.0 (3)
O1—C4—C5—C10	177.3 (3)	O2—C7—C8—O3	−1.8 (4)
C3—C4—C5—C10	−2.6 (4)	C6—C7—C8—C9	2.7 (5)
O1—C4—C5—C6	−1.5 (4)	O2—C7—C8—C9	178.9 (3)
C3—C4—C5—C6	178.6 (3)	O3—C8—C9—C10	178.2 (3)
C10—C5—C6—C7	1.8 (4)	C7—C8—C9—C10	−2.6 (5)
C4—C5—C6—C7	−179.4 (3)	C6—C5—C10—C9	−2.0 (5)
C5—C6—C7—C8	−2.2 (5)	C4—C5—C10—C9	179.3 (3)
C5—C6—C7—O2	−177.6 (3)	C8—C9—C10—C5	2.3 (5)
C11—O2—C7—C8	7.6 (4)	C7—O2—C11—O3	−10.4 (4)
C11—O2—C7—C6	−176.5 (4)	C8—O3—C11—O2	9.3 (4)

### Hydrogen-bond geometry (Å, °)

**Table:**

*D*—H···*A*	*D*—H	H···*A*	*D*···*A*	*D*—H···*A*
C9—H9A···O1^i^	0.93	2.53	3.209 (4)	130

Symmetry codes: (i) −*x*+1, *y*−1/2, −*z*+3/2.
